# Supplementary material for: The free energy landscape of retroviral integration
Source: Nat Commun. 2019 Oct 18;10:4738. doi: 10.1038/s41467-019-12649-w (PMC6802197; doi:10.1038/s41467-019-12649-w)
Supplement: Supplementary file 1 — Supplementary Information [file 41467_2019_12649_MOESM1_ESM.pdf]

Supporting Information for

The Free Energy Landscape of Retroviral Integration

Vanderlinden *et al.*

Content

Supporting Figure 1. AFM and gel electrophoresis resolve product topologies.

Supporting Figure 2. Modelling target DNA folding in TCCs and STCs.

Supporting Figure 3. AFM demonstrates auxiliary binding interfaces and geometries of TCCs.

Supporting Figure 4. Kinetic modelling of the target length-dependence of integration.

Supporting Figure 5. Changes in extension and extension fluctuations on intasome binding and unbinding.

Supporting Figure 6. Dynamic bridging and site selection in the TCC.

Supporting Figure 7. Supercoil relaxation dynamics in the STC.

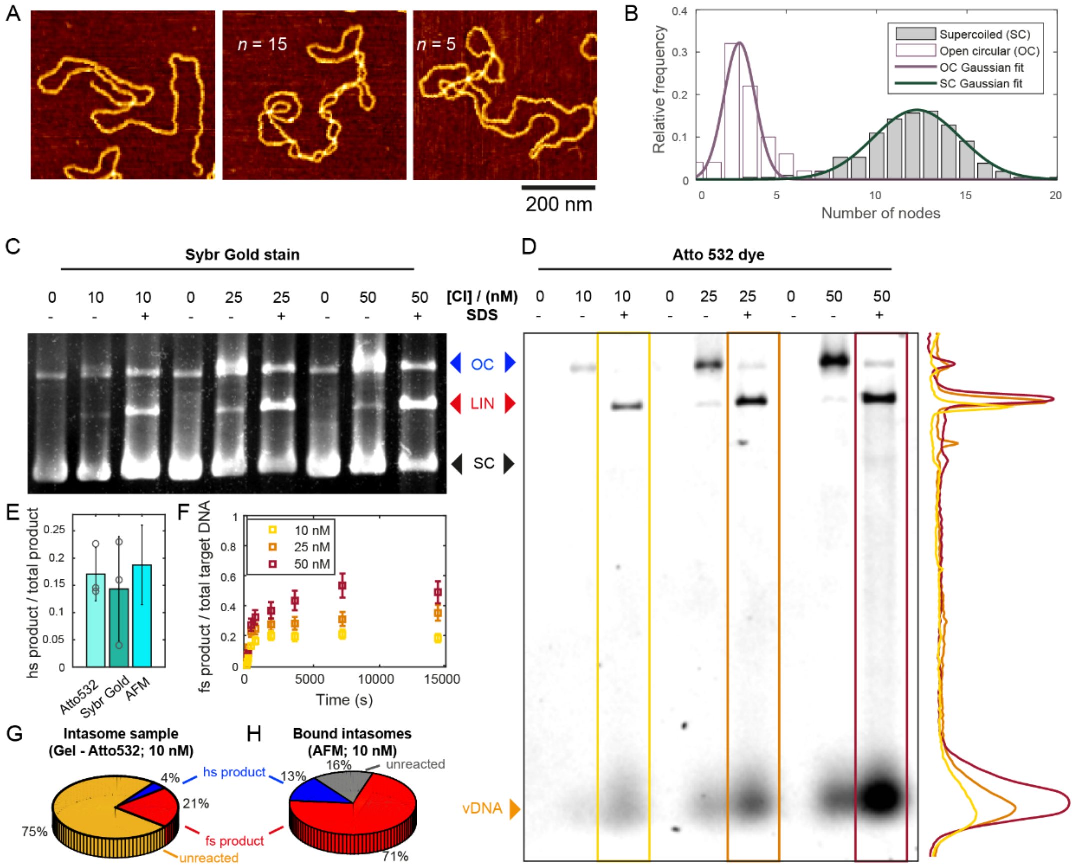

Supporting Figure 1. AFM and gel electrophoresis resolve product topologies. A. Example AFM images of linear (L), open circular (OC), and supercoiled (SC) plasmid molecules. **B.** Distributions of the number of intrachain crossings from AFM data on open circular (generated using Nt.BspQI nicking endonuclease) and supercoiled pBR322 plasmid molecules. For supercoiled pBR322, the node number distribution features a distinct peak, centered at 12.2 ± 3.4 nodes (mean ± SD; n = 211). The node number distribution of open circular pBR322 exhibits a peak centered at 2.4 ± 1.2 nodes (mean ± SD; n= 50). **C.** Gel electrophoretic separation of reaction mixtures obtained by incubating intasomes assembled with fluorescently labeled viral DNA mimetics and supercoiled pBR322 in reaction buffer. Untreated and chemically deproteinated samples are analyzed before and after chemical deproteination. The gel was imaged by staining total DNA using the stain Sybr Gold. **D.** Same gel as in (A) visualized by selective detection of the Atto532 dye attached to the unprocessed termini of the viral DNA mimetics. The free viral DNA mimetics ("vDNA") are visible as a fast-migrating band. Colored boxes (lanes 3,6, and 9) indicate the regions selected for generating the integrated intensity profiles shown on the right of the gel picture. **E.** Comparison of the ratio of half-site to total product, as determined through densitometric analysis of Atto532-label and Sybr Gold stain (mean ± SD over 3 different intasome concentrations) and by single molecule counting of AFM images (error is propagated $\sqrt{n}/n_{tot}$). The results from all three methods agree, within experimental error. **F.** Kinetics of full site integration in a supercoiled target, as assessed via densitometric analysis of Sybr Gold-stained gels. Error bars reflect approximate 10% uncertainty on the densitometric analysis of the gel bands. **G.** Determination of the yield of full-site and half-site integration product with respect to the total of viral DNA mimetics in the intasome sample, by densitometric analysis of Atto532 intensity profiles. **H.** Determination of the yield of full site and half site integration based on the topology of the target plasmid after deproteination of intasome-bound complexes observed by AFM imaging.

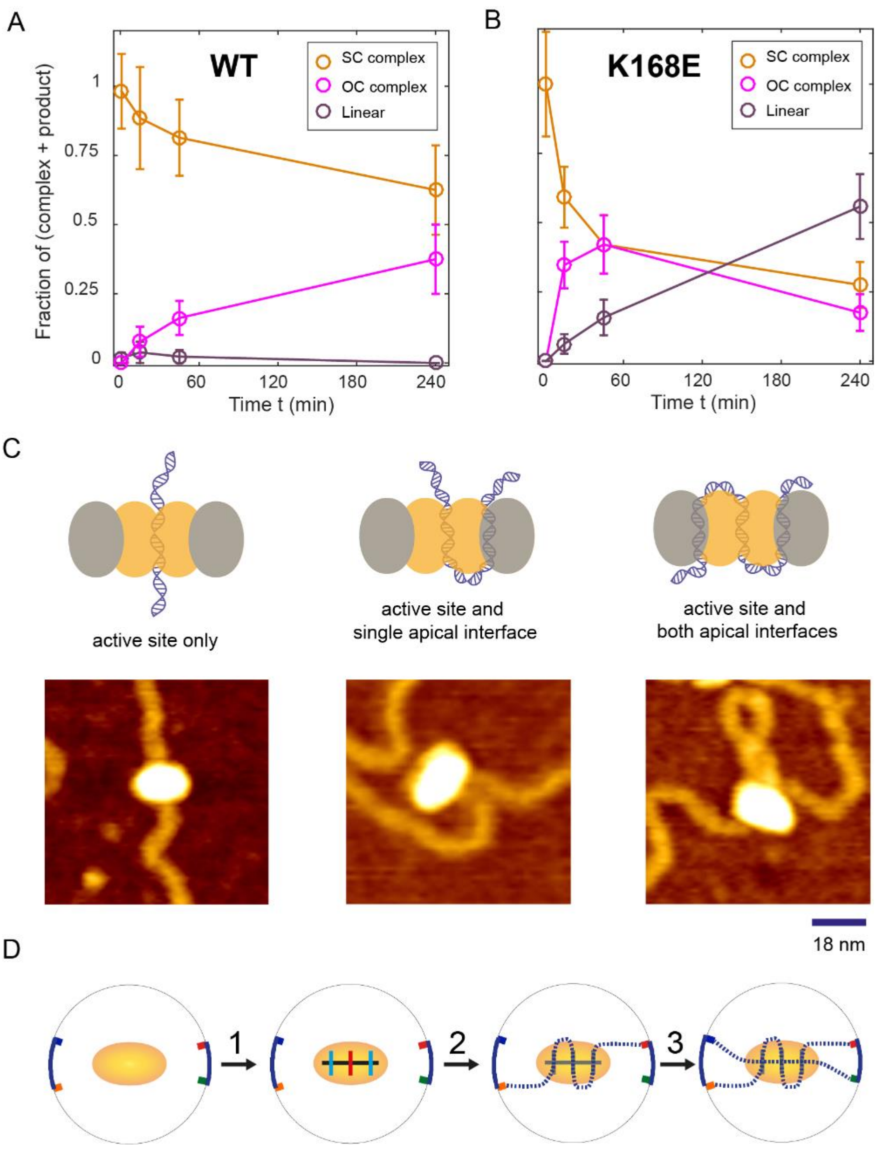

Supporting Figure 2. Modelling target DNA folding in TCCs and STCs A. Fractions (Error bars are $\sqrt{n}/n_{tot}$) of supercoiled (SC) complex, open circular (OC) complex, and linear products, with respect to the total of complexed and reacted plasmids in samples treated with wild type and **B.** K168E mutant intasomes which disassemble more rapidly. **C**. Further, AFM images of purified (see Methods) and partially disassembled wild type STC support an S-shaped fold of target DNA. Model and AFM images of (left) a STC bound solely to the active site, (middle) STC with engaged active site and a single apical interface, (right) STC with the active site and both apical interfaces engaged. **D.** Reconstruction of a mesoscale model for DNA folding in TCCs and STCs. Building on the experimentally determined exit angles (mean values are depicted as squares outside the intasome area) of target DNA loops (blue lines connecting the mean exit angle positions) extruding from intasomes assembled on supercoiled plasmid DNA, and (1) taking into account the 2D-projected positions of binding interfaces (apical: light blue, longitudinal: black, active site: red), we constrain our model using the knowledge that the target segment binding the active site additionally binds the apical auxiliary interfaces (2). The model can be completed by connecting the remaining entry- and exit-angle positions using the presence of the longitudinal auxiliary binding interface (3).

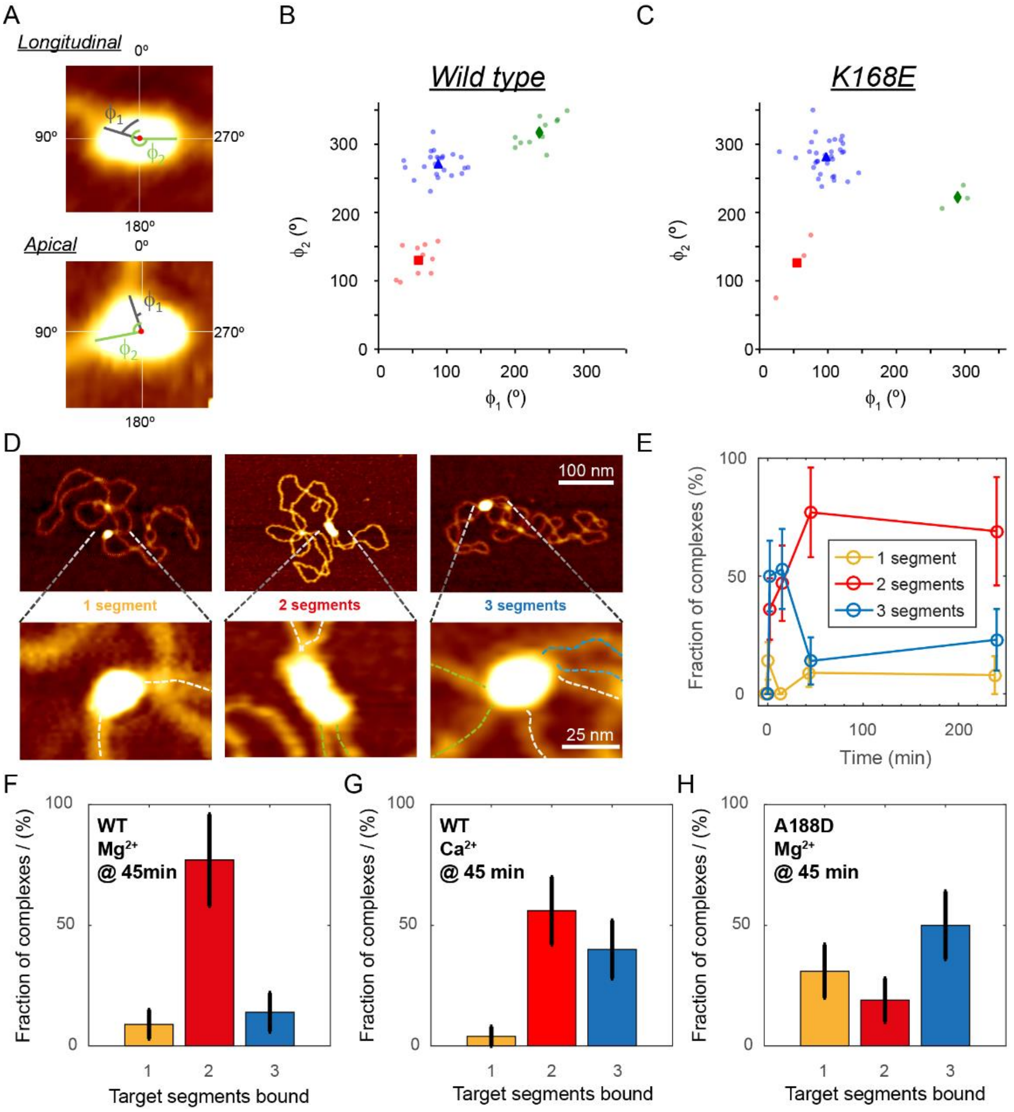

Supporting Figure 3. AFM demonstrates auxiliary binding interfaces and geometries of TCCs. A. Entry- and exit- angle determination from AFM topographs of intasomes. Topographs are subjected to a threshold of 1.5 nm and the resulting particle is rotated in-plane to align its long axis with the horizontal. The 2D-projected center of mass is determined and serves as the reference point for the angle determination. The angles are calculated with respect to the reference frames as indicated. The smaller angle is referred to as ϕ_1 and the larger angle ϕ_2. **B**. (ϕ_1, ϕ_2) scatter plot of wild type intasomes bound to a single target DNA segment, depicting the clusters reflecting the apical (green and red datapoints) and longitudinal complexes (blue datapoints). **C.** (ϕ_1, ϕ_2) scatter plot of K168E intasomes bound to a single target DNA segment, depicting the clusters reflecting the apical (green and red datapoints) and longitudinal complexes (blue datapoints). **D.** Representative AFM images of intasomes bound to a single, two, or three target DNA segments. **E.** Types of wild type intasome complexes bound to different number of target DNA segments as a function of incubation time. **F.** Number of target segments bound after 45 min of incubation of supercoiled pBR322 target DNA with wild type intasomes under reaction conditions, **G.** under conditions that impede reaction (buffer with Ca^{2+} instead of Mg^{2+}), and **H.** with A188D mutant intasomes under reaction conditions. Error bars are $\sqrt{n}/n_{tot}$.

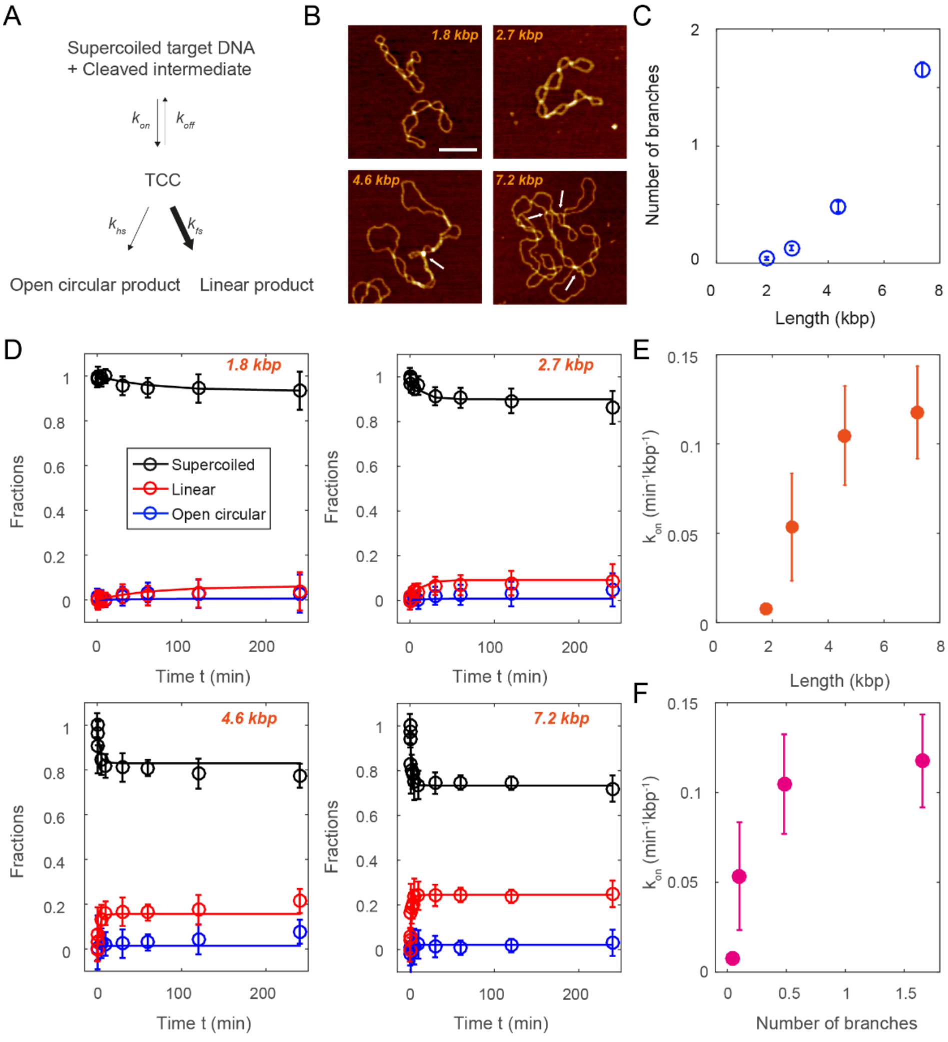

Supporting Figure 4. Kinetic modelling of the target length-dependence of integration. A. Minimal kinetic model wherein the rate for formation of a functional TCC k_{on} is target DNA dependent and strand transfer kinetics (full-site: k_{fs}; half-site: k_{hs}) are target DNA independent. The model assumes a DNA length-independent strand transfer yield (fraction of TCC converted to STC). The thicknesses of the arrows reflect the fitted rate constants. **B.** Representative AFM images of negatively supercoiled plasmids with a supercoiling density of −0.05 and varying lengths. White arrows indicate branch points (*i.e.* the points where the superhelix axes from more than two plectonemic segments intersect). Scale bar is 100 nm. **C.** Mean number of branch points as a function of plasmid DNA length determined by AFM. **D.** Data of ensemble strand transfer reactions using negatively supercoiled plasmids of different length as a target. Points and error bars are the mean and SD from 3 independent experiments. A global fit of the model depicted in (A) to the data ($\chi^2_{Red} = 0.20$) yields reaction rate constants $k_{hs} = 0.53$ min^{-1} and $k_{fs} = 5.96$ min^{-1}, a negligible off-rate ($k_{off} < 10^{-10}$ min^{-1}), and a DNA length-dependent on-rate k_{on}. **(E)** The rate constant for target capture k_{on}, normalized per kbp of target DNA increases with DNA length and **(F)** with the number of branch points of the target DNA and saturates as the number of branch points approaches one. The error bars in (E) and (F) are 95% CI on the fit parameter. Target capture is rate-limiting relative to strand transfer ($k_{hs} = 0.53$ min^{-1}; k_{fs}=5.96 min^{-1}). Source data are provided as a Source Data file.

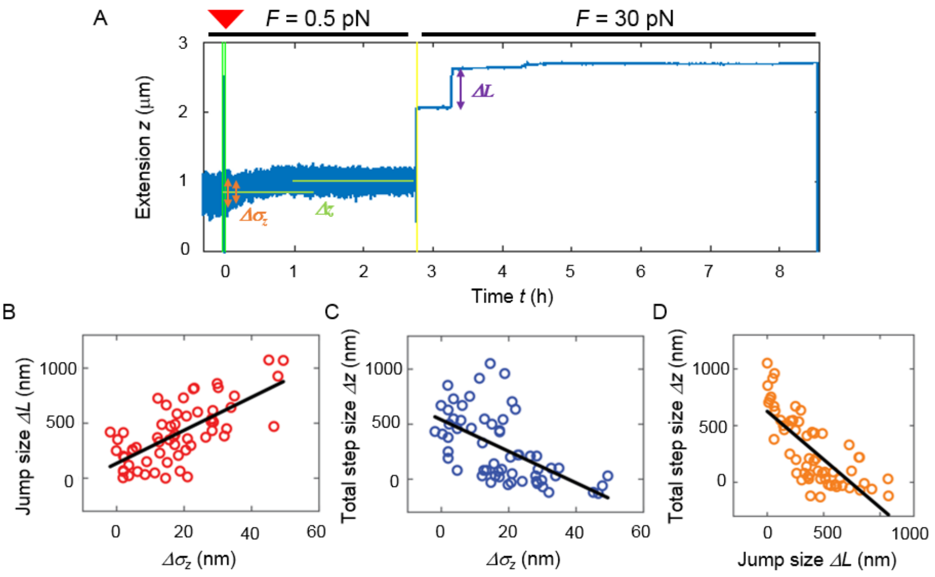

Supporting Figure 5. Changes in extension and extension fluctuations on intasome binding and unbinding. A. Extension time-trace of wild-type intasome interacting with a negatively supercoiled tether in magnetic tweezers, at low force, and on increasing molecular tension. The change in extension fluctuation $\Delta\sigma_z$ on initial binding, the total length increment on supercoil removal Δz, and the extension jump ΔL on forced dissociation are depicted. **B**. Correlation of the change in extension fluctuations $\Delta\sigma_z$ upon initial binding of intasomes and the size of the extension jump ΔL ("loop size") on forced dissociation at high force. The line is a linear fit (Pearson's correlation coefficient ρ = 0.695; p = 9.5·10^{-7}; p = 7.3·10^{-10}; slope = 15.0). **C.** Correlation of the change in extension fluctuations $\Delta\sigma_z$ upon initial binding of intasomes and the total supercoil removal after reaction Δz. The line is a linear fit (Pearson's correlation coefficient ρ = −0.584; p = 9.5·10^{-7}; slope = −14.3). **D.** Correlation of the extent of total supercoil relaxation Δz with the size of the extension jump ΔL on forced dissociation. The line is a linear fit (Pearson's correlation coefficient ρ = −0.749; p = 5.8·10^{-12}; slope = −0.85).The data are consistent with a model that assumes random binding and reaction of intasomes along the initial plectoneme as depicted schematically in Fig. 4A, which suggests a positive correlation between ΔL (which is a measure for the effective DNA length "pinched off" upon binding of the intasome along the plectoneme, *i.e.* the DNA length between the binding position and the plectoneme end loop) and $\Delta\sigma_z$. A clear positive correlation between ΔL and $\Delta\sigma_z$ is indeed observed (panel B). The fitted slope suggests a reduction of $\Delta\sigma_z$ by 1 nm for every 15 nm of effective DNA length sequestered upon binding. We note, however, that the relationship between $\Delta\sigma_z$ and plectoneme length is likely only approximately linear. The same random binding model suggests a negative correlation between the total DNA length released upon supercoil removal Δz (which is a measure of the effective DNA length between the intasome binding position and the origin of the plectoneme) and $\Delta\sigma_z$, as depicted in Fig. 4A. A clear negative correlation between Δz and $\Delta\sigma_z$ is indeed observed (panel C), with a slope that is approximately equal in magnitude and opposite in sign to the slope determined in A. Similarly, the model suggests a negative correlation between Δz and ΔL, as indeed observed experimentally (panel D).

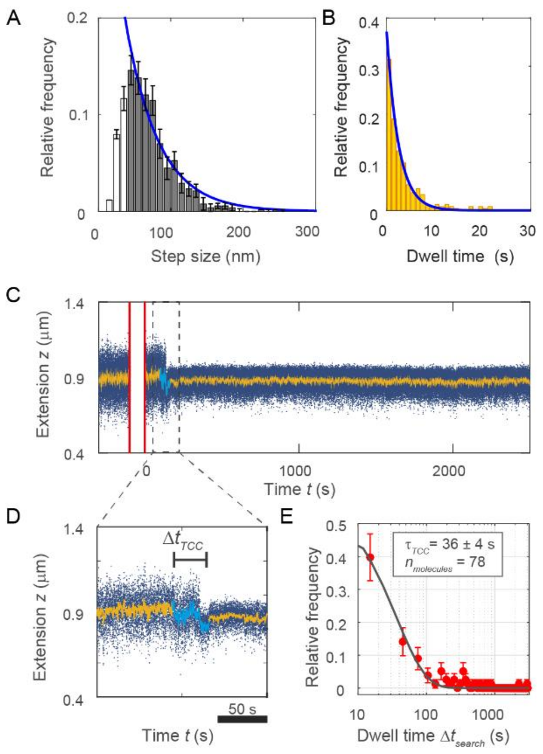

Supporting Figure 6. Dynamic bridging and site selection in the TCC. A. Distribution of step-wise changes in extension during the interaction of wild type intasomes with negatively supercoiled target DNA under non-reactive conditions (Ca^{2+}-buffer). Small step sizes (≤ 30 nm) of short duration are not detected by the step finding algorithm. Consequently, the corresponding bins are under-sampled. For larger step sizes, the distribution is continuous and well-described by an exponential fit with characteristic length ~ 45 nm. **B.** Dwell time distribution (n = 243) of the hopping dynamics obtained from extension time-traces of negatively supercoiled target with wt intasomes in reaction buffer in the presence of 10 µM Raltegravir. The distribution is well described by a single exponential with a mean lifetime τ^{acc}_{Ralt} = 2.7 ± 0.2 s. **C.** Dynamic bridging in the TCC is rapidly followed by strand transfer: representative time trace of extension z as a function of time for a supercoiled tether after injection of 10 nM of CI intasome (wild type), depicting the reduction of extension fluctuations characteristic of intasome-target interaction. Dynamic bridging (light blue) is followed by stabilization of extension hopping. **D.** Magnification of the boxed region in (C) and definition of the dwell time for dynamic bridging in the TCC Δt_{TCC}. **E.** Distribution of dwell times Δt_{TCC} (error bars are $\sqrt{n}/n_{tot}$) and fit using a single exponential decay with lifetime τ_{TCC} = 36 ± 4 s (error is 95% confidence). Source data are provided as a Source Data file.

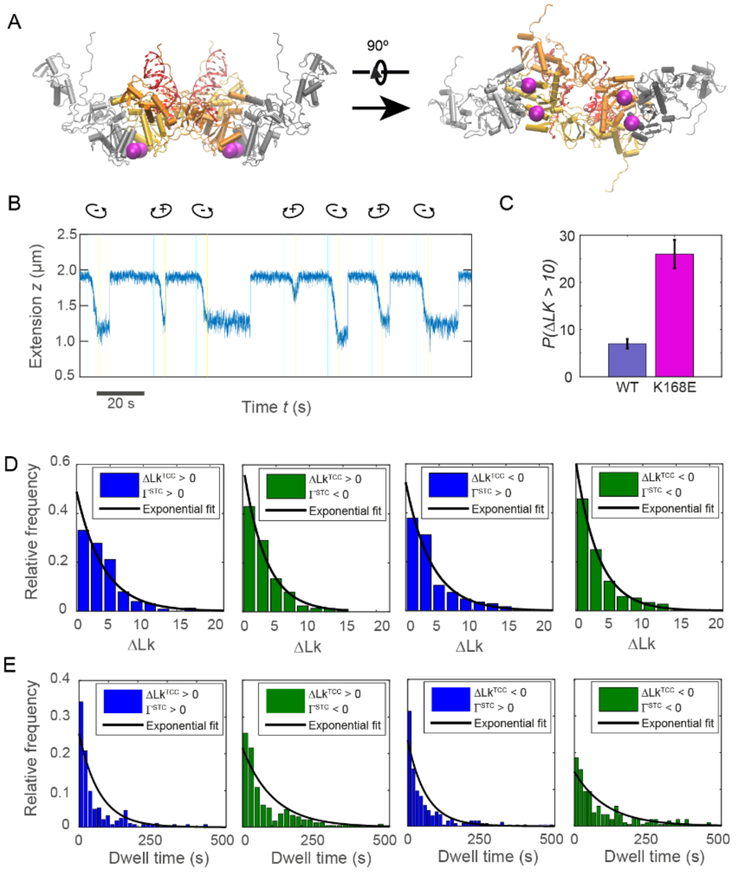

Supporting Figure 7. Supercoil relaxation dynamics in the STC. A. Atomistic model of PFV intasome refined against SAXS data, with residues K168 at the interface of the outer dimers highlighted as pink spheres. **B.** Typical time trace of supercoil relaxation events in STCs of K168E intasomes after repeated supercoil introduction by the external magnets. Magnet rotation results in variable extension changes due to long unbound states of the apical interfaces. Blue and yellow lines indicate start and end-point of the magnet rotation. **C.** Probabilities of large (*ΔLk* > 10) step sizes on relaxation of magnet-induced supercoils are significantly different ($p < 0.0001$; 2-sample t-test) for wild-type and K168E mutant intasomes. errors are $\sqrt{n}/n_{tot}$ **D.** Distributions of step sizes *ΔLk* on relaxation of supercoils in STCs; dependence on supercoil chirality of the target before and after strand transfer. From left to right: relaxation of positive supercoils in STCs formed on positively supercoiled target DNA (<*ΔLk*>= 4.00 ± 0.52; $n = 227$), relaxation of negative supercoils in STCs formed on positively supercoiled target DNA (<*ΔLk*>= 3.24 ± 0.40 ($n = 288$), relaxation of positive supercoils in STCs formed on negatively supercoiled target DNA (<*ΔLk*>= 3.83 ± 0.37; $n = 412$), relaxation of negative supercoils in STCs formed on negatively supercoiled target DNA (<*ΔLk*>= 3.39 ± 0.36; n =444). **E.** Distribution of dwell times Δt_{Apic}^{STC} on relaxation supercoils in STCs; dependence on supercoil chirality of the target before and after strand transfer. From left to right: relaxation of positive supercoils in STCs formed on positively supercoiled target DNA (τ_{Apic}^{STC} = 70.1 ± 8.2 s; $n = 227$), relaxation of negative supercoils in STCs formed on positively supercoiled target DNA (τ_{Apic}^{STC} = 105 ± 13.3 s; $n = 288$), relaxation of positive supercoils in STCs formed on negatively supercoiled target DNA (τ_{Apic}^{STC} = 62.8 ± 7.3 s; $n = 412$), relaxation of negative supercoils in STCs formed on negatively supercoiled target DNA (τ_{Apic}^{STC} = 110 ± 14.5 s; $n = 444$). Errors are 95% CI. Source data are provided as a Source Data file.
